# A novel curcumin analog inhibits canonical and non-canonical functions of telomerase through STAT3 and NF-κB inactivation in colorectal cancer cells

**DOI:** 10.18632/oncotarget.27000

**Published:** 2019-07-16

**Authors:** Seyung S. Chung, Pranabananda Dutta, Nathaniel Chard, Yong Wu, Qiao-Hong Chen, Guanglin Chen, Jaydutt Vadgama

**Affiliations:** ^1^ Division of Cancer Research and Training, Charles R. Drew University of Medicine and Science, Los Angeles, California 90059, USA; ^2^ Jonsson Comprehensive Cancer Center, UCLA, Los Angeles, California 90095, USA; ^3^ Department of Chemistry, California State University at Fresno, Fresno, California 93740, USA; ^4^ David Geffen School of Medicine, UCLA, Los Angeles, California 90095, USA

**Keywords:** curcumin analog, cancer stem cells, colorectal cancer, STAT3, NF-κB

## Abstract

Curcumin is a biologically active polyphenol that exists in Indian spice turmeric. It has been reported that curcumin exerted anti-inflammatory, anti-oxidant and anti-cancer effects in numerous *in vitro* and *in vivo* studies. However, it is not well-understood the molecular mechanism of curcumin for the cancer stem cells and telomerase in colorectal cancer. In this study, compound 19, a nitrogen-containing curcumin analog, was used to treat human colorectal cancer cells. Compound 19 showed a greater anti-proliferative activity than curcumin while displayed no significant toxicity toward normal human colon epithelial cells. Compound 19 exerted anti-inflammatory activities by deactivating STAT3 and NF-κB. In cancer stem cell populations, CD44, Oct-4 and ALDHA1 expressions were abolished upon treating with compound 19. Cancer stem cell biomarkers CD51 and CD133 positive populations were reduced and telomerase activities were decreased with the reduced STAT3 binding to hTERT promoters. This means compound 19 dually inhibits canonical and non-canonical functions of telomerase. Furthermore, compound 19 treatments induced cell cycle arrest at G1 phase and apoptosis. Human apoptosis-related array screening revealed that activated caspase 3, catalase, clusterin and cytochrome C led to apoptosis. Taken together, our data suggest that compound 19 can be a novel therapeutic agent for metastatic colorectal cancer by concurrently targeting STAT3 and NF-κB signaling pathways.

## INTRODUCTION

Colorectal cancer (CRC) is the third leading mortality in the United States and fourth leading cause of cancer-related death globally [1]. CRC mortality is primarily due to the metastatic progression, with the liver being the most frequent organ of metastatic colonization [2]. The 5-year survival rate of patients with CRC metastasis is less than 10% [3]. Current treatments of CRC include surgery and chemotherapy [4]. Standard chemotherapy of FOLFOX (5-Flurouracil, leucovorin and oxaliplatin) has been recommended for colon cancer patients at the stages II and III [5]. Unfortunately, patients frequently develop drug resistance against chemotherapy and metastasis leading to death within 5 years of diagnosis [6]. Thus, there are significant unmet needs for the novel therapeutic regimen that can better treat CRC patients at a low risk of recurrence.

Curcumin is a substance in turmeric and known to have multiple biologically beneficial effects [7]. A number of *in vitro* and *in vivo* studies revealed that curcumin has anti-cancer effects including colorectal cancer [8]. It has been reported that curcumin inhibited cancer cell proliferation, induced the cell cycle arrest and apoptosis in various cancer types [9]. Curcumin has also been extensively investigated for their potential to stop metastasis from occurring [10, 11]. However, actual mechanisms of curcumin on the telomerase and stemness in cancer cells are poorly defined. Therefore, we tested a nitrogen-containing novel curcumin analog, compound 19, with the potential to simultaneously inhibit cancer stemness and telomerase in human colorectal cancer cells.

Several curcumin analogs were developed and studied. Recently, Madan and associates reported that curcumin analog HO-3867 converted mutant p53 to wild-type p53 and increased cytotoxicity on the cancer cells [12]. It has been shown that another curcumin analog L48H37 induced apoptosis through ROS-mediated endoplasmic reticulum stress and STAT3 pathway in human lung cancer cells [13]. Liang and colleagues synthesized a new mono-carbonyl curcumin analog and induced G2/M cell cycle arrest and mitochondria-mediated apoptosis in colorectal cancer cells [14]. Nonetheless, to date, there is no curcumin analog that showed both selective cancer stem cell inhibition and efficient telomerase activity reduction. We herein demonstrate the novel curcumin analog that inhibits cancer stem cell phenotype and telomerase in colorectal cancer cells.

Metastasis is driven by the variant cancer cells that acquired invasiveness, migration and colonization during the cancer development [15]. Mounting evidence suggests that these metastatic variant cells has cancer stem cell (CSC) like phenotype [16]. Cancer stem cells are unique cell populations that can differentiate and generate cancer cells in various types of cancer including colorectal cancer [17]. Collective work has revealed that cancer stem cells contribute to the therapeutic resistance and metastasis, leading to the recurrence in patients [18]. For these reasons, cancer stem cells are becoming a potential target for anti-cancer therapy. Although cancer stem cells are believed to be a promising target for novel therapies, the specific mechanisms by which these putative therapeutics could intervene is elusive.

One of the main characteristics of cancer stem cells is the self-renewal through the capability of modulating different molecular signaling pathways, including Wnt/β-Catenin, Sonic Hedgehog and Notch pathways [19]. Pharmacological targeting cancer stem cell specific signaling might be an excellent modality for colorectal cancer therapy. In cancer stem cells, transcription factors selectively activated include signal transducer and activator of transcription 3 (STAT3) and nuclear factor κB (NF-κB) [20, 21]. STAT3 is a latent transcription factor that conveys various signals from cytokines and growth factors from cell membrane to nucleus [22]. Tyrosine 705 of STAT3 gets phosphorylated (pSTAT3) upon various cytokines and growth factors stimulation which leads to the subsequent transcriptional activation of its target genes [23]. STAT3 is aberrantly activated in the aggressive cancers, hence a poor prognosis factor [24]. Another transcription factor frequently activated in cancer is NF-κB [25]. NF-κB regulates the cancer cell survival and drug resistance, simultaneously contributes to immune response to inflammation [26, 27]. In this study, we used pSTAT3 and pNF-κB as inflammation biomarkers as well as targets for cancer stem cells to study the novel synthetic curcumin analog for its anti-cancer effects.

Telomerase elongates the telomere DNAs at the end of chromosomes [28]. It is composed of reverse transcriptase (TERT) and RNA component (TERC) and activated up to 90% of human malignancies including CRC, as targeting telomerase or hTERT structure has been suggested for cancer therapy [29]. Besides its canonical function for telomeric extension, hTERT has been implicated for cancer stem cell generation and maintenance through the cellular reprogramming processes [30, 31]. Activated telomerase contributes to the metastasis via this conversion from cancer cells to cancer stem cells. This non-canonical function of telomerase attracted more attention to target telomerase for concurrently inhibiting cancer stem cell phenotype and telomerase in colorectal cancer therapeutic approaches.

We have previously shown that combined treatments with curcumin and green tea polyphenol epigallocatechin gallate (EGCG) inhibited cancer stem cell phenotype in the aggressive breast cancer [32]. Herein, we hypothesized that a nitrogen-containing curcumin analog might inhibit cancer stem cell phenotype and telomerase through its anti-inflammatory impacts. We report a novel synthetic curcumin analog’s inhibition on cancer stem cell phenotype and telomerase activity by inactivation of STAT3 and NF-κB in colorectal cancer stem cells. Further, we show that G1-phase cell cycle arrest and increased apoptosis with a novel curcumin analog challenge. Our data suggest that a novel curcumin analog compound 19 can be an efficient therapeutic regimen for the advanced colorectal cancer.

## RESULTS

### Compound 19 has a greater cytotoxicity than curcumin towards colorectal cancer cells while displayed a low toxicity on the normal epithelial colon cell line

**Figure 1:**
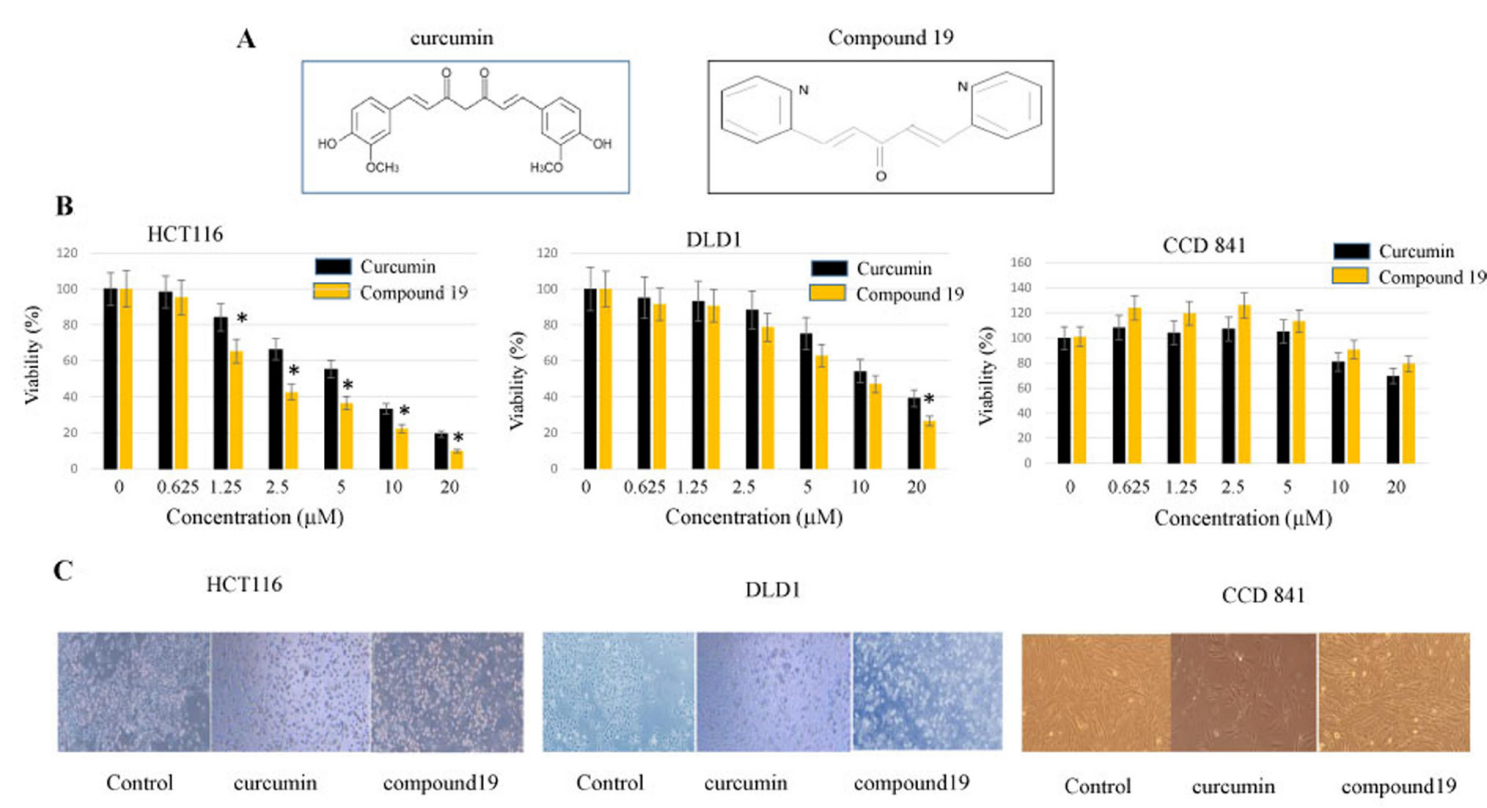
Anti-proliferative effects of curcumin and compound 19 on the human colorectal cancer cell lines HCT116 and DLD1. (**A**) Chemical structures of curcumin and its analog compound 19 (**B**) Colorectal cancer cell line HCT116 was treated with curcumin and compound 19 at 0~20 mM for 48 hours (Left panel). Another cancer cell line DLD1 (middle panel) and normal colon cell line CCD841 (right panel) were also treated with curcumin and compound 19 for 48 hours at 0 ~ 20 mM. Error bars represent standard deviation. (**C**) HCT116 (left panel), DLD1 (middle panel) and CCD 841 (right panel) were treated with curcumin and compound 19 monitored for the morphological changes.

A nitrogen-containing curcumin analog, compound 19, was synthesized with a central monoketone linker and two terminal pyridine rings (Figure 1A). We first tested the cytotoxicity of compound 19 on colorectal cancer cell lines. The HCT116 and DLD1 cancer cells were treated with compound 19 alongside its parental curcumin. Curcumin and compound 19 were serial diluted from 20 μM to 0.625 μM concentrations and monitored for cell viability with MTS assay as described in the methods. As shown in Figure 1B, the IC_50_ values of compound 19 and curcumin against HCT116 cells were 2.13 μM and 7 μM, respectively. In the DLD1 cell model, the IC_50_ values of compound 19 and curcumin were 10 μM and 12 μM, respectively. Interestingly, compound 19 did not suppress 50% cell proliferation up to 20 μM in a CCD 841 normal colon epithelial cell line, suggesting a low toxicity of compound 19 on the normal colon cell line. The difference in drug response is possibly due to the different genetic make-up of two cell lines. In HCT116, there is a mutation in RAS while there is a p53 mutation in DLD1 [33]. Both HCT116 and DLD1 cell lines showed apoptotic morphology when treated with compound 19 for 48 hours (Figure 1C). However, compound 19 did not induce CCD841normal colon cell death. Taken together, compound 19 demonstrates a greater and more selective cytotoxicity against human colorectal cancer cells compared to curcumin.

### Compound 19 challenge abolished expressions of cancer stem cell biomarkers in the colorectal cancer stem cell populations

**Figure 2:**
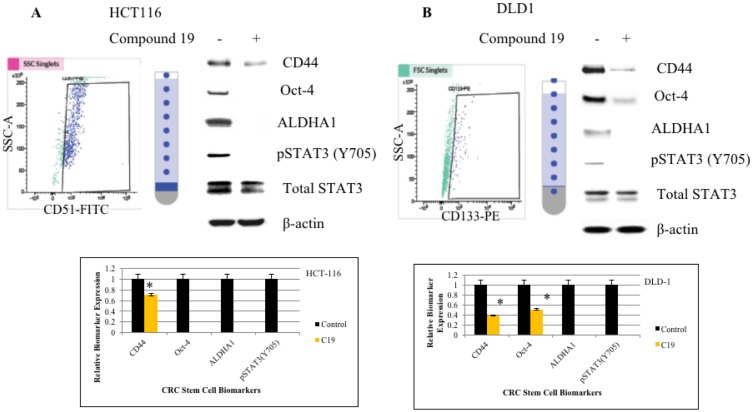
FACS cell sorting of HCT116 and DLD1 and western analyses of cancer stem cell biomarkers. HCT116 and DLD1 were stained with CD51-FITC and CD133-PE, respectively. Sorted cells were applied to FACS cell sorting for cancer stem cell isolation. (**A**) HCT116 cells were sorted out for its CD51 (+) subpopulations (top left panel). Sorted cells were treated with compound 19 at 10 mM for 48 hours and subject to western analyses for cancer stem cell biomarkers (top right panel). Protein band intensities were quantified and presented in graphs (bottom panel). (**B**) DLD1 cells were sorted out for CD133 (+) subpopulations (top left panel). Sorted cells were treated with compound 19 at 10 mM for 48 hours and subject to western analyses for cancer stem cell biomarkers (top right panel). Protein band intensities were quantified and presented in graphs (bottom panel).

Next, we wished to test whether compound 19 can inhibit cancer stem cell phenotype in the colorectal cancer cells. To this end, we first sorted out cancer cells for cancer stem cells based on the biomarker positivity. For the HCT116 cell line, we sorted out CD51 (+) and CD51 (–) cells and for the DLD1 cell line, cells were sorted out based on the CD133 (+) and CD133 (–) populations (Figure 2A and 2B). CD51 (+) HCT116 cells expressed CD44, Oct-4, ALDHA1 and STAT3 was activated as shown by phosphorylation of Tyrosine 705. CD44 is a cancer stem cell biomarker and Oct-4 and ALDHA1 are stem cell biomarkers. When we treated CD51 (+) cells with compound 19, CD44 expression was downregulated and Oct-4, ALDH1 and pSTAT3 expressions were abolished. Similarly, in DLD1 CD133 (+) cells, CD44, Oct-4, ALDHA1 were expressed and STAT3 was activated. Compound 19 treatment downregulated CD44 and Oct-4 and abolished ALDHA1 expression. Our data suggest that the curcumin analog has the specific inhibitory effects against cancer stem cell phenotype in the colorectal cancer cells.

### Compound 19 showed anti-inflammatory effects by inhibiting NF-κB and STAT3 activation in the cancer stem cell populations

**Figure 3:**
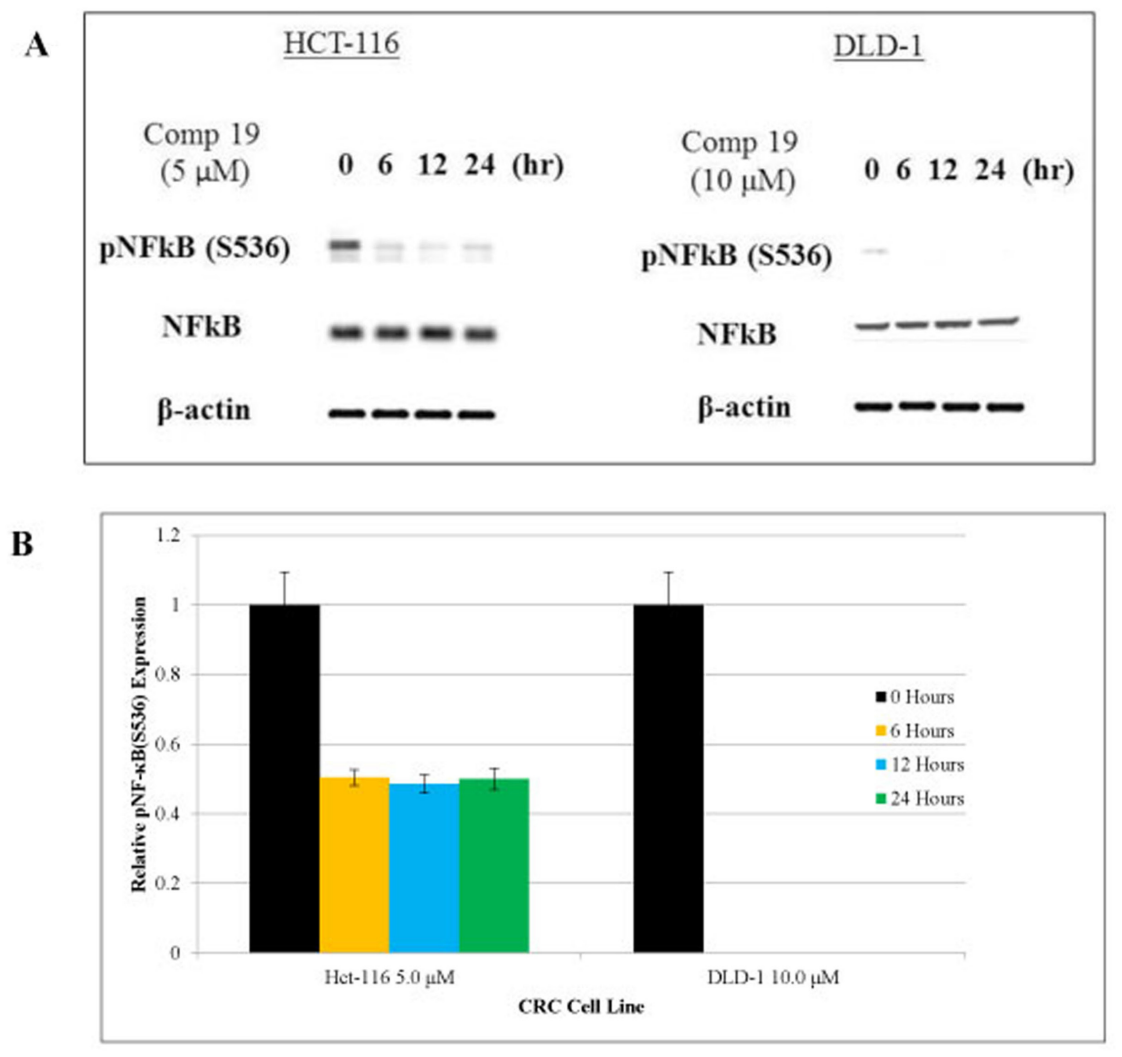
Phosphorylated NF-κB was inhibited by compound 19 in colorectal cancer cell lines. HCT116 and DLD1 were treated with compound 19 on a time course of 24 hours. (**A**) HCT116 and DLD1 treated with compound 19 were analyzed for phosphorylated NF-κB (S536) for 24 hours. Compound 19 showed inhibitory effects in a time-dependent manner. (**B**) pNF-κB relative expression levels were presented quantitatively. Protein bands were measured by image J software and error bars represent standard deviations.

Nuclear factor-κB (NF-κB) is a transcription factor that plays a key role in immune response, inflammation, cell growth, survival and development [34]. Aberrant NF-κB activation contributes to development of various autoimmune disease and inflammatory disease [35]. Cancer stem cell populations were sorted out by CD51 (+) and CD133- (+) and we found that both NF-κB and STAT3 are phosphorylated in the colorectal cancer stem cell population (Figures 2 and 3A). Therefore, inhibiting NF-κB and STAT3 signaling has potential therapeutic applications for cancer stem cells. As shown in Figure 3A, compound 19 showed the time-dependent inhibitory effects on the pNF-κB (S536). Western blot of pNF-κB bands were measured the intensity and graphed with Image J software analyses (Figure 3B). Serine 536 is phosphorylated by IKKbeta and is critical for activation of canonical NF-κB pathway [36]. Our cell sorting data suggest that both NF-κB and STAT3 are activated in the cancer stem cell populations. More importantly, curcumin analog compound 19 showed a specific anti-cancer stem cell phenotype with the anti-inflammatory effects on human colorectal cancer cells.

### Compound 19 treatments decreased cancer stem cell populations from colorectal cancer cells

**Figure 4:**
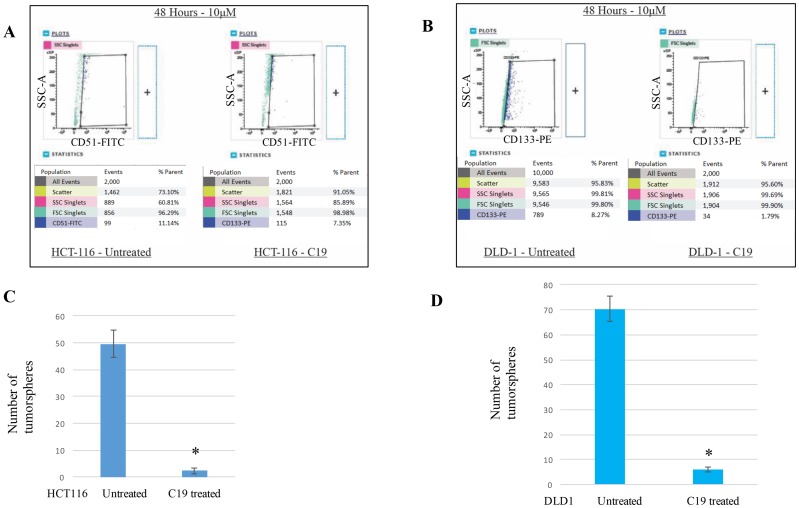
Cancer stem cell biomarker CD51 and CD133 positive subpopulations were decreased with compound 19 treatments. To determine the anti-cancer stemness effects of compound 19, HCT116 and DLD1 cells were treated with compound 19 for 48 hours, then applied to flow cytometry analysis for CD51-FITC and CD133-PE. (**A**) HCT116 cells were treated with compound 19, then applied to flow cytometry analyses. (**B**) DLD1 cells were treated with compound 19, then applied to flow cytometry analyses. (**C**) HCT116 cells were pre-treated with compound 19 for 24 hours, then applied to tumorsphere formation assay. (**D**) DLD1 cells were pre-treated with compound 19 for 24 hours, then applied to tumorsphere formation.

Since molecular-level stem cell markers are clearly expressed in the cancer stem cell populations, we wished to test the “cell-level” stem cell maker changes with the compound 19 treatments. HCT116 and DLD1 cells were treated with 10 μM compound 19 for 48 hours and examined for CD51 (+) and CD133 (+) population changes, respectively. In HCT116, CD51 (+) populations were reduced from 11.14% to 7.35% when compared to control (Figure 4A). We have performed two more independent experiments to examine the cancer stem cell population changes. Untreated HCT116 showed 12.37 + 1.15 % and compound 19 treated HCT116 showed 6.52 ± 1.12 %. In DLD1 cell line, CD133 (+) cell populations were also decreased from 8.27% to 1.79% when compared to untreated control (Figure 4B). With two more independent experiments, CD133 (+) populations were decreased from 9.16 ± 0.92 % to 2.11 ± 0.28 %.

As a functional analysis of tumorigenicity, we carried out the tumorsphere formation assays. HCT116 and DLD1 were pre-treated with compound 19 and applied to matrigel semi-solid microenvironment. Tumorspheres were counted after 7 days of culture. HCT116 untreated control formed 49 ± 5 spheres and compound 19 treated cells formed 2.3 ± 1.1 spheres (Figure 4C). Similarly, DLD1 untreated control shaped 70.3 ± 5.1 spheres but treated cell formed 6 ± 1 spheres (Figure 4D). Our data suggest that curcumin analog compound 19 has a specific anti-cancer stem cell phenotype effects on both molecular and cell-levels.

### Compound 19 challenge inhibited STAT3 binding to hTERT promoter, decreased telomerase activity and shortened telomeric DNAs

**Figure 5:**
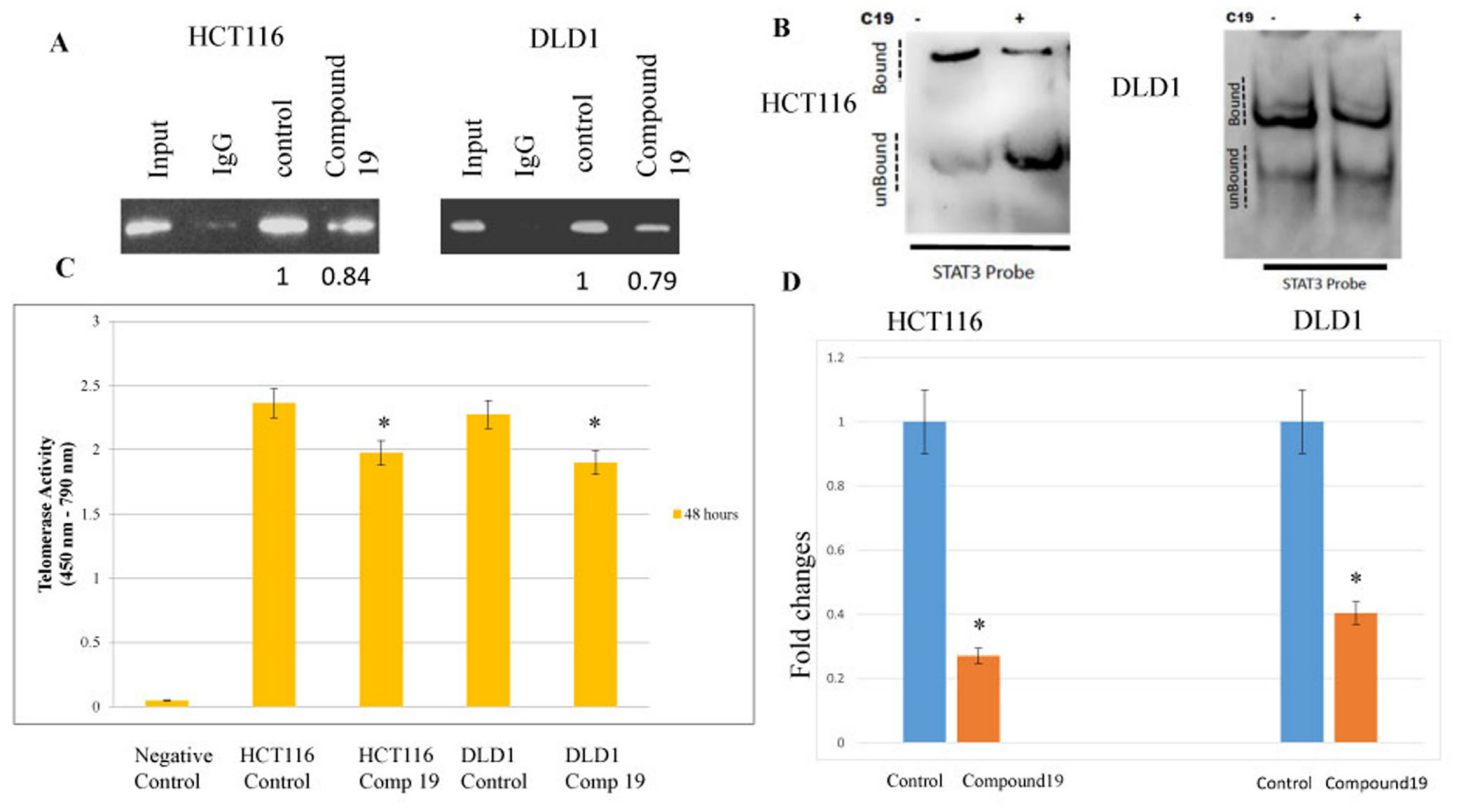
STAT3 binding to promoter region hTERT were inhibited by the compound 19 and corresponding telomerase activities were decreased and telomeric DNAs were shortened. Transcription factor STAT3 binding to hTERT promoter region was tested with chromatin immunoprecipitation assay. (**A**) Both HCT116 and DLD1 were treated with compound 19 and applied to ChIP assay. Densiometric values of DNA bands were measured with Image J soft ware with the untreated control cell set as 1.0. (**B**) EMSA assay was performed with compound 19 treated HCT116 and DLD1. (**C**) Telomerase activities were measured by TRAP PCR reaction conjugated to Elisa assay. (**D**) HCT116 and DLD1 cells were treated with compound 19 and applied for telomere length quantification qPCR assays.

The findings that compound 19 inhibited STAT3 activation suggest telomerase activity may be decreased due to the reduced STAT3 binding to its promoter. Therefore, we wished test whether STAT3 binding has been inhibited by compound 19 treatments and subsequently decreased the telomerase activities in the colorectal cancer cells. It was shown that consensus STAT3-binding sites (TTCNNNGAA) reside within the hTERT promoter [37]. We performed the chromatin immunoprecipitation (Chip) assay to monitor the STAT3 binding to hTERT promoter region with or without compound 19 treatments. STAT3 binding was reduced with compound 19 treatments in both HCT116 and DLD1 cell lines (Figure 5A). To confirm the compound 19 specific effects on STAT3, we performed the electrophoretic mobility shift assay (EMSA). Compound 19 challenge decreased the STAT3 binding to its target DNA probes in both HCT116 and DLD1 (Figure 5B). In agreement with this result, telomerase activities were decreased with compound 19 challenges. In HCT116 cells, telomerase activity was decreased from 2.36 to 1.97 units by PCR-Elisa assays (Figure 5C). Similarly, telomerase activities were reduced from 2.27 to 1.90 in DLD1 cells. We also measured the telomere length with the compound 19 treatments with qPCR assays. To this end, we used the telomere specific primers and performed qPCR assays. As shown in Figure 5D, compound 19 treated HCT116 cells showed the telomere length decrease from 1 to 0.27 folds compared to the control while treated DLD1 showed the telomere length decrease from 1 to 0.40 folds compared to control. Our data suggest that compound 19 has a specificity to STAT3 protein and inhibit the STAT3 binding to hTERT promoter and decrease the telomerase activities in the colorectal cancer cells.

### Compound 19 and curcumin treatments induced specific cell cycle arrest in colorectal cancer cells

**Figure 6:**
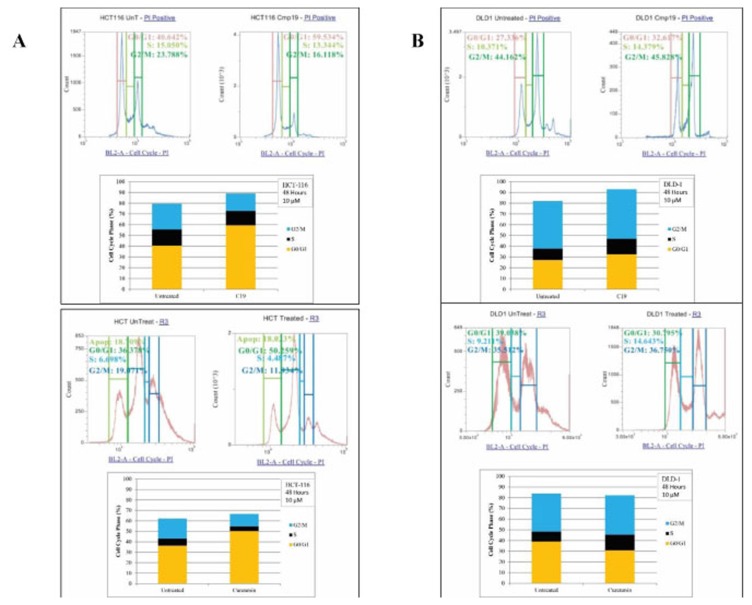
FACS analysis of cell cycle of HCT116 and DLD1 treated with compound 19 (10 mM). HCT116 and DLD1 were treated with compound 19, then applied to FACS analyses for cell cycle profiling. (**A**) Representative images of cell cycle distribution of cancer cell line HCT116 with compound 19 at 10 μM for 48 hours treatments (top panel) and with curcumin at 10 mM for 48 hour treatments (bottom panel) (**B**) The distribution of cell cycle of DLD with compound 19 at 10 μM for 48 hours treatments (top panel) and with curcumin at 10 mM for 48 hour treatments (bottom panel).

We then studied the effect of compound 19 and curcumin on cell cycle profiles. We treated HCT116 and DLD1 cells with the compound 19 and curcumin for 72 hours. The cancer cells were then stained with hypotonic staining buffer and applied to flow cytometry. In HCT116 control, G0/G1 phase was 40.64%, S phase was 15.05% and G2/M phase was 23.79% (Figure 6A). Upon compound 19 treatments, G0/G1 phase has increased to 59.53% whereas G2/M phase has decreased to 16.12 %. Similarly, curcumin treatments induced G0/G1 phase increase from 36.38 % to 50.26 % and S phase decrease from 19.07% to 11.93 %. The DLD1 cell cycle profile was distinct from that of HCT116. DLD1 control showed 27.34 % of G0/G1 phase, 10.37% of S phase and 44.16 % of G2/M phase profiles (Figure 6B). When treated with compound 19, G0/G1 phase increased to 32.62 %, S phase increased to 14.38 % and G2/M phase increased to 45.83 %. DLD1 showed concurrent increase in G0/G1 and S phases. However, when DLD1 was treated with curcumin, only S phase has been increased from 9.21 % to 14.64 % (Figure 6B). Our data suggest that compound 19, like its parent compound curcumin, induced the specific cell cycle arrest in the human colorectal cancer cells.

### Colorectal cancer cell apoptosis induced by compound 19

**Figure 7:**
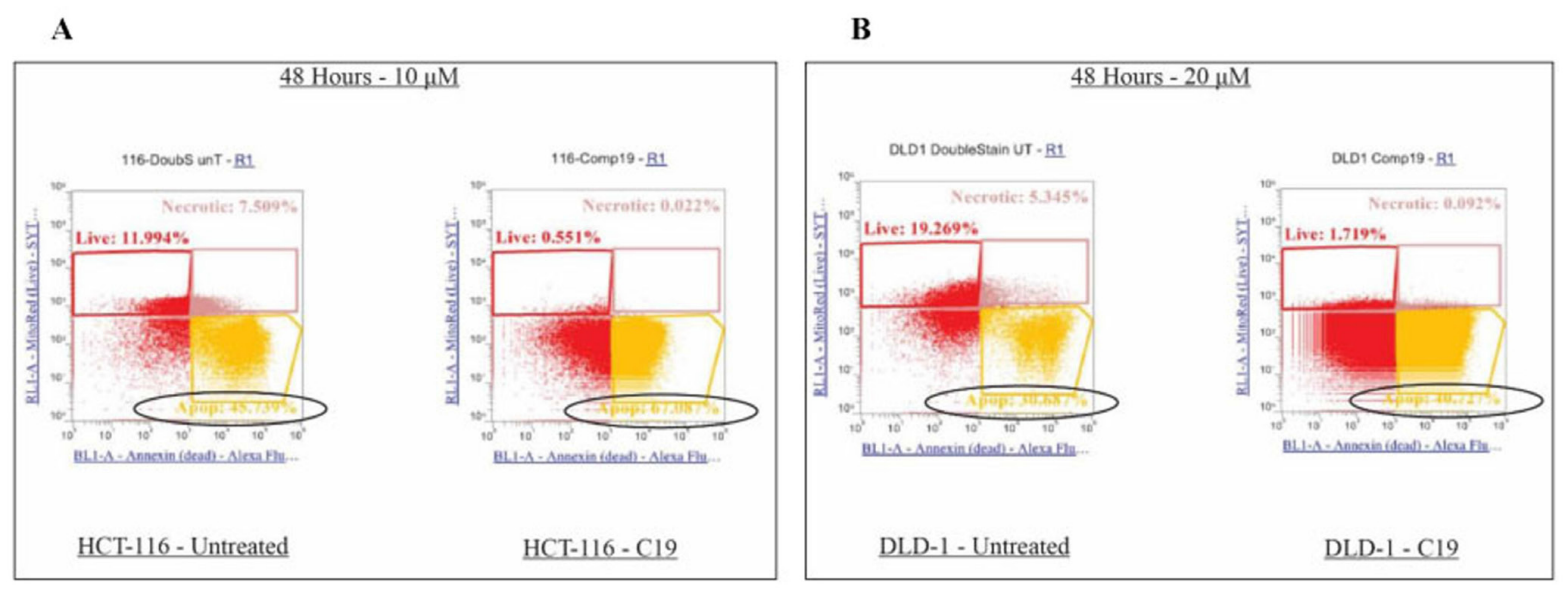
Effects on apoptosis upon compound 19 treatments in the colorectal cancer cell lines. Apoptosis was increased upon compound 19 treatments. (**A**) Apoptotic profile of HCT116 with compound 19 treatments. Apoptosis was measured with mitotracker and annexin V staining for mitochondrial depolarization and serine externalization, respectively. (**B**) Apoptotic profile of DLD1.

Since specific cell cycle arrest was induced, further examination was performed on the colorectal cancer cell apoptotic induction by compound 19. Specifically, compound 19 treated HCT116 and DLD1 cells were applied to mitochondrial potential/annexin V apoptosis analyses as described in Methods and Materials. In this assay, apoptotic populations show yellow fluorescence with decreased red fluorescence (mitotracker ^low^/annexin V+), which present a decreased mitochondrial potential and phosphatidyl serine translocation. As shown in Figure 7, HCT116 control showed 45.74 % in the quadrant of mitotracker ^low^/annexin V+ and compound 19 treatment increased apoptosis to 67.09 % (Figure 7A). Similarly, in DLD1 cells, control showed 30.69 % and compound 19 treatment increased the apoptosis to 40.73 %. Our data suggest that compound 19 efficiently induce apoptosis in human colorectal cancer cells.

### Compound 19 activated caspase-3 apoptotic pathways in colorectal cancer cells

**Figure 8:**
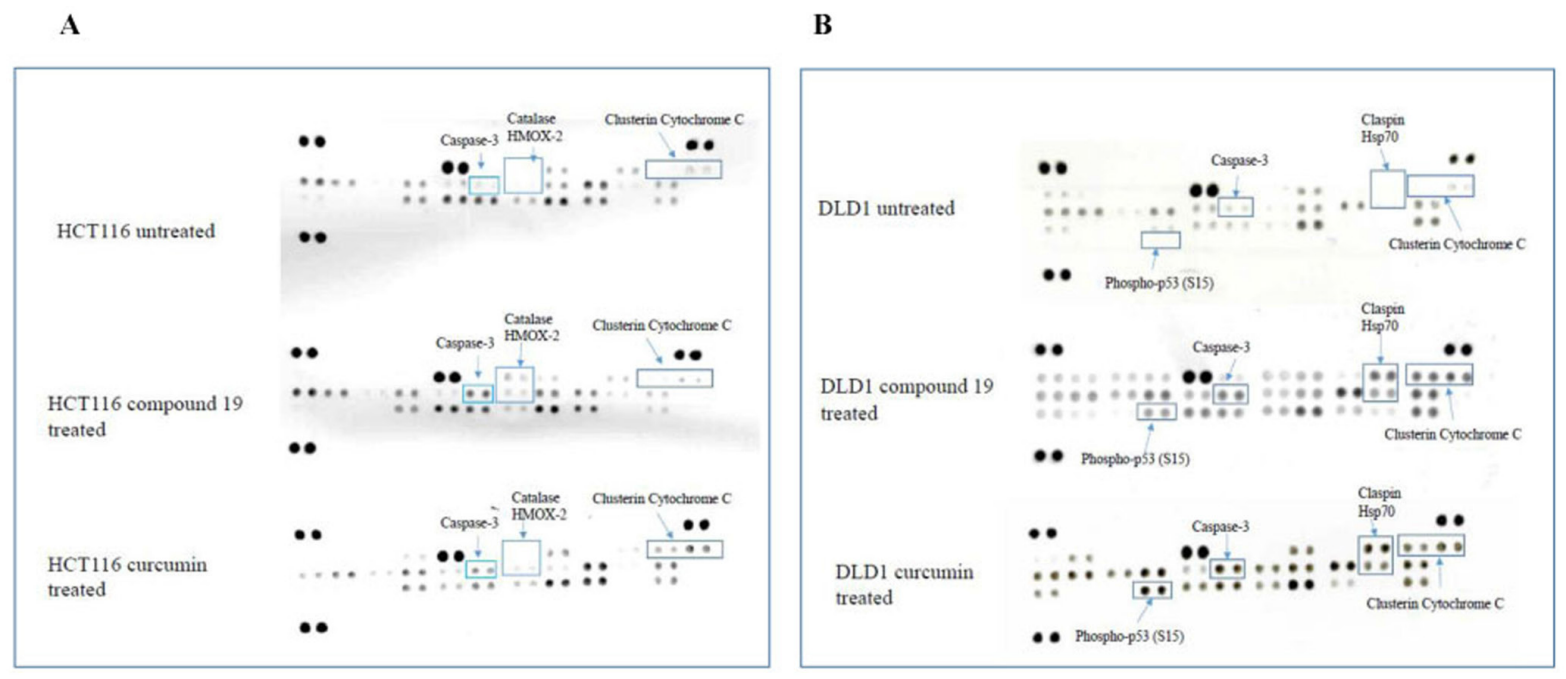
Human apoptosis array analyses of colorectal cancer cell lines treated with compound 19 and curcumin. (**A**) HCT116 was treated with compound 19 at 10 μM for 48 hours (middle array) and curcumin 10 μM for 48 hours (bottom array) and applied for the apoptosis arrays. (**B**) DLD1 cells were treated with compound 19 10 μM for 48 hours (middle array) and curcumin 10 μM for 48 hours (bottom array) and applied for the apoptosis arrays.

Colon cancer cell apoptosis was clearly activated by compound 19. Hence, we wished to examine the specific apoptotic pathways that compound 19 turned on when treated to the colon cancer cells. To this end, human apoptosis arrays were used to detect relative expression levels of 35 apoptosis-related proteins simultaneously. The HCT116 and DLD1 cells were treated with the compound 19 and curcumin, and then applied to the apoptosis arrays to uncover activated proteins. As shown in Figure 8, in HCT116 cells, caspase 3 was cleaved; catalase and HMOX2 were activated upon compound 19 treatments (Figure 8A). Caspase 3 exists as an inactive proenzyme that undergo proteolytic process to produce two subunits that dimerize to form active enzyme [38]. Caspase 3 cleavage plays a key role in cell apoptosis progression [39]. Catalase promotes the decomposition of hydrogen peroxide to water and oxygen whereas HMOX2 is a heme oxygenase that cleaves heme to form biliverdin [40, 41]. However, when treated with curcumin, HCT116 showed clusterin and cytochrome C activation. Clusterin is a molecular chaperon that helps folding of secreted protein and cytochrome c is released from mitochondria to cytosol to activate a caspase cascade [42, 43]. In DLD1, phospho-p53 (S15), claspin and Hsp70 were activated upon compound 19 and curcumin treatments (Figure 8B). P53 activates DNA repair proteins when DNA has been damaged [44]. Claspin is an essential upstream regulator of checkpoint kinase 1 and Hsp70 protects cells from thermal or oxidative stress [45, 46]. Claspin and Hsp70 activation is unique in compound 19 treatments and it is distinct from other cancer cell line HCT116. Our data suggest that compound 19 can induce distinct apoptotic pathways during apoptosis.

### Identification of differential gene expression upon compound 19 treatment in colon cancer cells using RNA-sequencing

**Figure 9:**
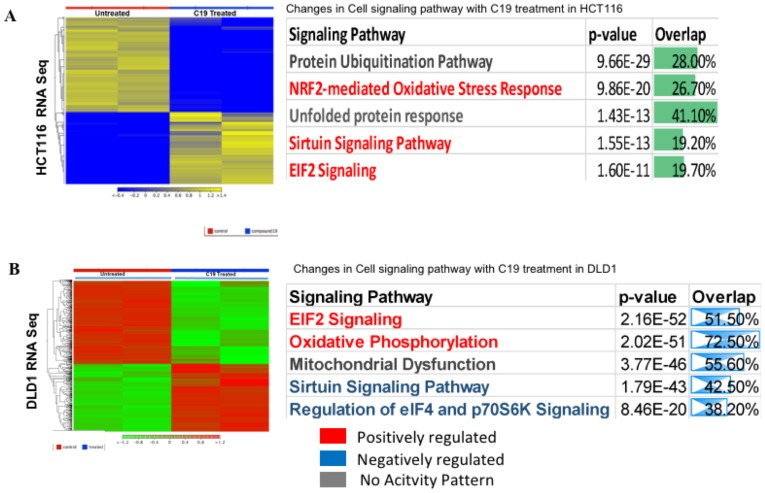
RNA-sequencing heat map and ingenuity pathway analysis. (**A**) HCT116 was treated with compound 19 and applied to RNA-seq analysis. Heat map and IPA pathways are presented. (**B**). DLD1 was treated with compound 19 and applied to RNA-seq study. Heat map and IPA pathways are presented. The whole IPA pathways and Gene Ontology (GO) data of HCT116 are presented as Supplementary Tables 1 and 2, respectively. The whole IPA pathways and Gene Ontology data of DLD1 are presented as Supplementary Tables 3 and 4, respectively.

We prepared RNA from untreated and compound 19 treated HCT116 and DLD1 and performed RNA-sequencing on mRNA in duplicate samples. Heat maps of whole transcriptome were presented for significantly differentially expressed genes in HCT116 untreated and compound 19 treated samples (Figure 9A). Ingenuity pathway analysis data are also presented. Top three pathways were protein ubiquitination pathway, NRF2-mediated oxidative stress response and unfolded protein response pathway. Thus, compound 19 might be affecting overall protein stability under redox stress in colon cancer cells resulting in apoptosis and cell cycle arrest as observed previously (Figures 6 and 7). Whole Ingenuity pathway analysis (IPA) data are presented in the Supplementary Materials (Supplementary Table 1). We have attached the gene ontology (GO) data set as Supplementary Table 2. The same RNA-sequencing analysis was carried out with DLD1. The heat map and IPA pathways were presented (Figure 9B). Complete IPA pathways and GO analysis data are presented as Supplementary data set (Supplementary Tables 3 and 4, respectively).

## DISCUSSION

**Figure 10:**
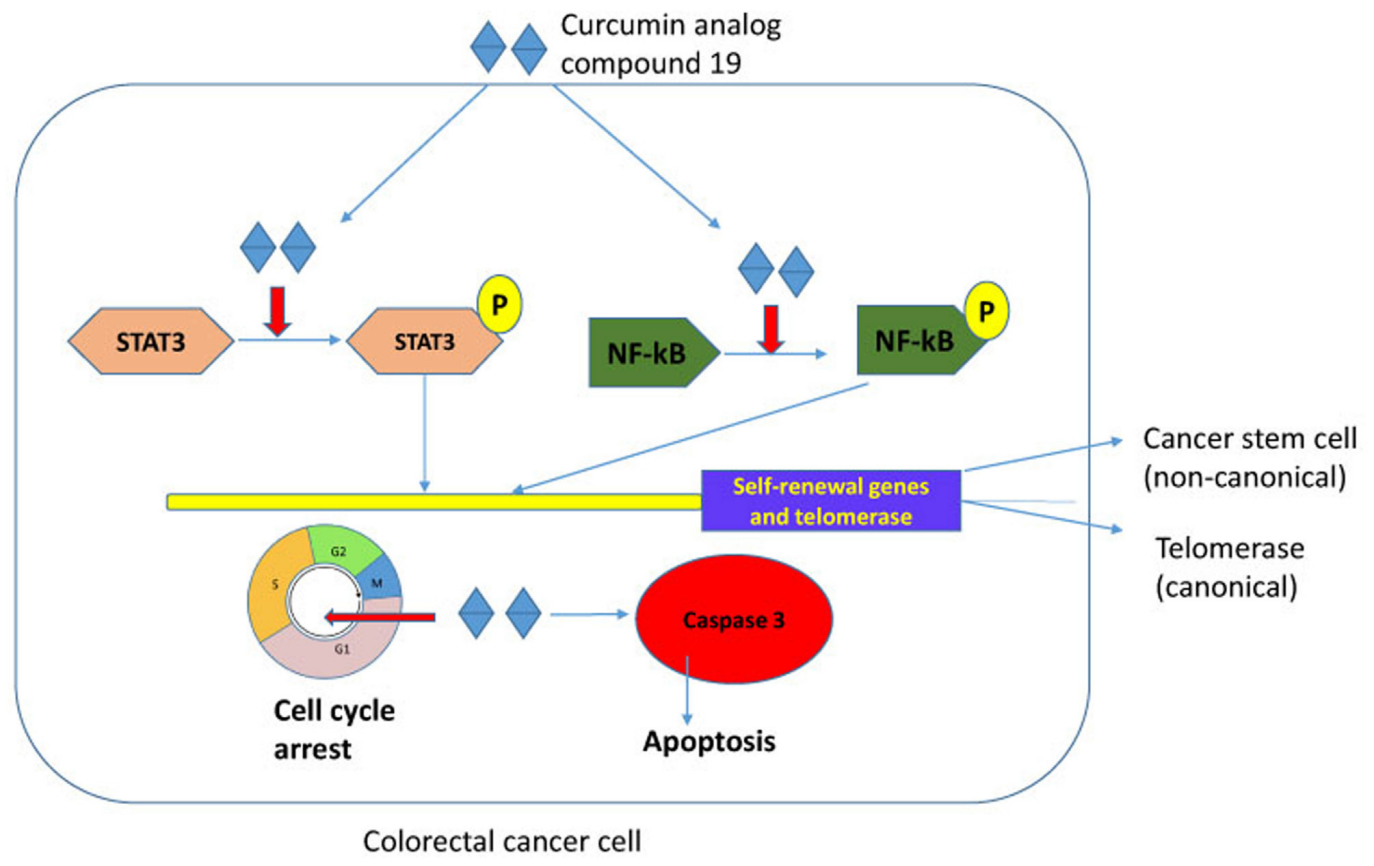
Schematic representation of compound 19 in colorectal cancer cells. Compound 19 concurrently inhibits STAT3 and NF-κB transcription factors in colorectal cancer. Both STAT3 and NF-κB transcriptionally activate self-renewal genes and telomerase that resulted in enhanced stemness. Compound 19 also arrested cell cycle and induced apoptosis in the caspase-3 activated pathway. Our model suggests that a novel curcumin analog efficiently inhibits cancer stemness by deactivating STAT3 and NF-κB, and consequently decreasing telomerase activity and downregulating self-renewal gene expressions.

Curcumin is a polyphenol and a major component of tumeric. It has many beneficial biological effects and exerts anti-oxidant, anti-inflammatory and anti-cancer activities (reviewed in [47]). However, the low bioavailability and a broad spectrum of targets have hampered its application in the clinical setting. Curcumin has been tested in clinical trials for its safety at high doses and activity against pancreatic cancer in human patients despite its low bioavailability [48]. The anti-tumor properties demonstrated by curcumin have led to our study its potential to target cancer stem cells and telomerase. Here, we have tested the synthetic curcumin analog compound 19 for its anti-cancer stem cell phenotype and anti-telomeric activities in colorectal cancer cells. Our experimental results indicate that compound 19 showed improved cytotoxicity on colorectal cancer cells, inhibited cancer stem cell phenotype, decreased telomerase activity and induced apoptosis efficiently. Both STAT3 and NF-κB were deactivated by compound 19 which resulted in reduced self-renewal gene expressions and telomerase activities (Figure 10).

Cancer stem cell concept present a new paradigm for cancer treatments. Cancer stem cells are cancer cells that have stem-like traits such as self-renewal and pluripotency [49]. It consists of small fraction of populations and often display chemo-resistance and radio-resistance during therapy, hence it is necessary to treat these cells to decrease metastasis and reduce recurrence. Compound 19 reduced the CD51 (+) subpopulation of HCT116 and CD133 (+) cells of DLD1 which are considered cancer stem cells. Compound 19 treatments also deactivated pSTAT3 and pNF-κB. Both STAT3 and NF-κB are transcription factors selectively activated in cancer stem cells [50]. Our data suggest that a novel curcumin analog might target STAT3 and NF-κB and consequently downregulate stem cell self-renewal gene expression transcriptionally. The strength of compound 19 lies in the ability to target STAT3 and NF-κB concurrently. It is still feasible that compound 19 interacts with the upstream signaling pathways of STAT3/NF-κB, thereby impacts the cancer stem cell phenotype. It remains to be seen if there is an interaction between compound 19 and potential upstream signaling pathways in colorectal cancer cells.

Telomerase is reverse transcriptase complex that elongates telomeres at the ends of chromosomes. It is overly activated in most cancers and now considered a universal biomarker for cancer [51]. In addition to its telomeric DNA polymerization, telomerase regulates a diverse cellular functions during oncogenesis [52]. Notably, telomerase reverse transcriptase plays a role in a transcriptional modulation of stemness genes in embryonic stem cells and cancer cells [53]. This “non-canonical” function of telomerase has attracted our attention as a target for therapy during the development of novel regimens for colorectal cancer. Herein, we tested the novel curcumin analog’s inhibitory effects on cancer stemness and telomerase side by side. STAT3 mediated transcriptional activation of hTERT led to self-renewal genes turn-on, hence increased the stemness in cancer cells. Compound 19 reduced cancer stem cell populations and deactivated STAT3 and NF-κB. Transcription factor STAT3 binding to hTERT promoter was decreased, consequently telomerase activity was reduced with compound 19 treatments. Our data suggest that compound 19 can be an efficient therapy for cancer stem cells by inhibiting STAT3 and repressing non-canonical function of telomerase for stemness in colorectal cancer. These results demonstrates the dual inhibition of compound 19 to stemness and telomerase which resulted in the cumulative effects on the metastatic cancer cells.

Apoptosis was clearly induced by compound 19, but unlike curcumin, it induced catalase and heme oxygenase in the HCT116 cell line. Curcumin and compound 19 showed the same apoptotic profiles in DLD1 cells. Structure wise, compound 19 has the similar two terminal aromatic rings, but is characteristic of the basic, nitro-containing pyridine rings. The molecular targets of curcumin as well as compound 19 have not been defined. The bioavailability of compound 19 is expected to be greatly improved compared with curcumin, due to the presence of metabolically stable mono-ketone linker and the nitrogen-containing aromatic rings, which will be measured in due course. Finally, more animal studies using curcumin and its analog are warranted and under way. Taken together, our data suggest that the nitrogen-containing curcumin analog compound 19 is a promising novel therapeutic regimen based on its anti-cancer stemness for the colorectal cancer.

The novel curcumin analog inhibits cancer stem cell phenotype in colon cancer cells. Human telomerase reverse transcriptase was transcriptionally suppressed by STAT3 inactivation upon the curcumin analog challenge. Furthermore, cell cycle was arrested and specific and distinct apoptotic pathways activated upon the curcumin analog treatments. Taken together, our data suggest that the novel curcumin analog, compound 19, can be a promising therapy for metastatic colorectal cancer.

## MATERIALS AND METHODS

### Cell lines and reagents

HCT116 and DLD1 colon cancer cell lines were purchased from the American Type Culture Collection (ATCC, Manasas, VA, U.S.A.). HCT116 and DLD1 cancer cells were maintained in a monolayer culture in DMEM/F12 (Dulbecco’s modified Eagle medium) with 10% fetal bovine serum, 2.5% L-Glutamine and 0.5% Penicillin/Streptomycin. Curcumin was purchased from Sigma-Aldrich (catalog number: C1386). Compound 19 was chemically synthesized by Dr. Qiao-Hong Chen’s lab at the Department of Chemistry, California State University Fresno. Both curcumin and compound 19 stock solutions were made at 10 mM in a dimethyl sulfoxide.

### Cell viability assay

Colon cancer cells were cultured in 96-well plates (2 × 10^4^ cells per well) at 37° C in a 5 % CO_2_ incubator. Cells were then treated with a gradient of concentrations of curcumin and compound 19. Cell viability was determined using the 96 Aqueous One Solution Cell Proliferation Assay kit [MTS,3-(4,5-dimethylthiazol-2-yl)-5-(3-carboxymethoxyphenyl)-2-(4-sulfophenyl)-2H-tetrazolium] (Promega, catalog number: G3580, Madison, WI, USA) following the manufacturer’s instructions. The quantity of formazan product was determined by measuring absorbance at 450 nm using a Promega Glo Max-Multi detection system (Promega, Madison, WI). IC50 concentration of compound 19 was determined by the dose-response experimentation.

### Western blot analysis

Monolayer cultures of respective cell lines at 80-90% confluence were lysed using 100 μl of RIPA buffer (Thomas Scientific Inc. Swedesboro, NJ). Tris-glycine (Bio-Rad, Irvine, CA) pre-cast gels were loaded with 50-100 μg of cell lysates. After electrophoresis, the gel was transferred to a nitrocellulose membrane for 1 hour. The membrane was blocked for 1 hour in 5% skim milk at 4° C. The membrane was then washed 3 times with 1x TTBS and incubated overnight with the primary antibody at 4° C. Primary antibodies of CD44, Oct-4, ALDHA1, STAT3, pSTAT3, pNF-κB, NF-κB and β-actin were purchased from Cell Signaling Technology (Danvers, MA). After incubation with the secondary antibodies conjugated with horseradish peroxidase (HRP), the protein bands were developed with the chemiluminescent reagents.

### Cell sorting and analyses

Cancer cells were sorted based on cancer stem cell biomarker positivity. For HCT116, CD51 (+) positive cells were sorted through the cell sorter (BD melody cell sorter, BD404XD, San Diego, CA, U.S.A) and for DLD1, CD133 (+) positive cells were sorted. Cancer cells were detached from the culture flask and transferred to 15 ml test tubes. For HCT116, 5 μl of CD51-FITC antibody was added to the tubes, and cells were incubated in the 4° C for 30 min. Cells were spun at 1,000 rpm for 5 min and washed with 1X PBS buffer. The cells were run through the BD cell sorter to profile and acquire the cancer stem cell population data. We used CD133-PE for the DLD1 cell line and performed the cell sorting as described above.

### Tumorsphere formation assay

Matrigel (Corning, catalog number: 356234), 200 μl was spread as a thick layer on a 24-well plate and allowed to polymerize at 37° C for 15 minutes. Cells (2 × 10^4^) grown as monolayer were trypsinized to single cells and plated on top of the pre-coated matrigel. Plates were incubated at 37° C to allow cells to fully settle before the mdia was replaced with appropriate culture media containing 5% matrigel. Cell were grown for 7 days. Images of representative fields were taken at the day 7. Tumorspheres > 100 μm were counted for the assay.

### Chromatin immunoprecipitation assay

Chromatin immunoprecipitation (ChIP) Assay Kit (Millipore, Catalog number: 17-295) was employed to study STAT3 binding to hTERT promoter region. DLD-1 or HCT116 cells were incubated with 1% formaldehyde for 20 minutes at 37° C. Cells were collected, lysed, sonicated, and incubated with 4 μg of antibodies to STAT3 overnight. PCR was used to amplify DNA bound to the immunoprecipitated histones after reversing the histone-DNA cross-links. Primer sets were designed flanking the possible STAT3 binding regions. Primer sequences: *hTERT* promoter primer sequence 1, forward primer 5ʹ-CCAAACCTGTGGACAGAACC-3ʹ and reverse primer 5ʹ-AGACTGACTGCCTCCATCGT-3ʹ and *hTERT* promoter primer sequence 2, forward primer 5ʹ-GGGGTGTCTTCTGGGTATCA-3ʹ and reverse primer 5ʹ-AAGGGCTGTGTTTGTGAATTG-3ʹ. PCR products of Chip assays were resolved on a 2.5% agarose gels. The STAT3 bound band densities were quantified by using Image J.

### Electophorectic mobility shift assay

Cell lysate after treatment was prepared using Signosis nuclear extraction kit (SK-0001). Equal amount of nuclear lysates from treated or untreated cells were mixed with biotinylated STAT3 or NFκB probes from Signosis EMSA assay kit. After 30 mins incubation at 22° C, samples Were run on a 6.6% polyacrylamide gel using 0.5x Tris Borate buffer (TBE). Samples with bound probes Were transferred onto a nitrocellulose membrane. The membrane subjected to UV irradiation On a Biorad GS gene linker with 125 milli Joules for 2 minutes. Immobilized probes were incubated With Steptavidin-HRP and were detected suing chemiluminiscence substrate. Image was obtained on a Licor Odyssey FC machine.

### Telomerase activity assays

Cancer cells were processed according to the manufacturer’s protocol for the TeloTAGGG Telomerase PCR ELISA kit (Roche, Orange, CA. Catalog number: 11854666910). Briefly, cell pellets were thawed in lysis reagent, incubated on ice for 30 minutes, and centrifuged at 16,000 *g* for 20 minutes at 4° C. Telomerase activity was immediately measured in the resultant supernatant using the telomeric repeat amplification protocol in which telomerase, if present in the cell lysate, adds telomeric repeats to the 3′ end of a biotin-labeled synthetic P1-TS primer. Samples were amplified by polymerase chain reaction (PCR), with P1-TS and P2 primers creating an elongated telomere. The PCR product was denatured and hybridized to a digoxigenin-labeled probe that detects telomeric repeats in a subsequent enzyme-linked immunosorbent assay (ELISA). Telomerase assays were performed three times independently and P values less than 0.05 were considered statistically significant.

To measure the telomeric DNA length changes, we used telomere length quantification qPCR assay kit (ScienCell, Catalog number: 8908, Carlsbad, CA, U.S.A.). Cells were treated with compound 19 and genomic DNAs were extracted and served as templates. Telomere specific primers were used for the qPCR. The 32 cycles of 95° C for 20 sec, 52° C for 20 sec and 72° C for 45 sec PCR reactions were performed. After qPCR is done, we used the qPCR instrument’s data analysis software for data analysis.

### Cell cycle analysis

Cell cycle profiles were analyzed using a flow cytometric DNA method. Briefly, cells were treated with curcumin and compound 19 for 48 hours. Cells were harvested, then incubated with 0.5 ml of hypotonic staining buffer (sodium citrate 0.25 g, Triton-X 100 0.75 ml, propium iodide 0.025 g, ribonuclease A 0.005 g and distilled water 250 ml) for 15 minutes or for a maximum of 1 hour before acquisition on the flow cytometer. The cells were acquired and analyzed with Attune NxT flow cytometer (Thermo Fisher Scientific, Chino, CA, U.S.A.).

### Analysis of apoptosis profiles and arrays

Mitochondrial potential and phosphatidyl serine externalization were evaluated using Mitochondrial Membrane Potential Apoptosis kit with Mitotracker TM Red and Annexin V Alexa Fluor 488 (Thermo Fisher Catalog number: V35116) following the manufacturer’s instructions. The cells were treated with resveratrol and 5-FU alone and in combination for 72 hours and processed with the Apoptosis kit and analyzed with the Attune NxT flow cytometer.

Human Apoptosis Array Kit was purchased from the R&D systems (Catalog number ARY009, Minneapolis, MN, USA). 1 x 10^7^ cells were rinsed with 1X PBS and solubilized with 1X lysis buffer provided in the kit. The apoptosis antibody membrane was placed in the 4-well multi-dish. 2 ml of array blocking buffer was added onto each membrane. The membrane was incubated for 1 hour on a rocking platform. After the incubation, the blocking buffer was removed. The antibody membrane was then washed three times with the 1X array wash buffer and 1.5 ml of cell lysates were placed on the membrane. The membrane was incubated for overnight at 4 degrees Celsius on an orbital shaker. The membrane was then washed with 20 mL of 1X array wash buffer and continued incubation on the orbital shaker for 5 minutes at room temperature. 2 ml of streptavidin-HRP solution was added onto the membrane. It was incubated for one hour at room temperature on an orbital shaker. Next, three washes were performed with 1X array wash buffer. The membrane was washed and treated with Lumi Glo and peroxide. The Bio-rad Gel Documentation System (Bio-Rad, catalog number 170–8195, Hercules, CA, USA) was used to take detailed pictures of the array using the Quantity One software using the Chemi Doc XRS function.

### RNA sequencing analysis

#### Illumina RNA seq library preparation

Total RNA was isolated after treatment and Quantified using Agilent Bioanalyzer RNA 600 Chip. RNA utilized for sequencing had RIN (RNA integrity Number) >= 8. Illumina. Illumina TruSeq stranded mRNA kit (Catalogue: 20020594) was used to prepare RNA Sequencing Libraries. Libraries were run on Bioanalyzer DNA 1000 chip to assess quality. Library quantitation was done using qubit 4.0.

#### Bioinformatics analysis

FASTQ files from sequencer run was analyzed using CLC Genomics Workbench 12 (https://www.qiagenbioinformatics.com/products/clc-genomics-workbench) (API version:12.0.1 Build number: 20181122013502 Build date: 1811220135 Build rev: 185754). Default parameter was utilized for mapping reads to Hg19 human genome (Ensembl GRCH37 v74). For every sample, TPM (Transcripts per Million) data for genes were generated after mapping to genome. Differential expression of genes between control and experimental samples were generated using EDGER statistical method in CLC Genomics Workbench. Heatmaps were generated with differentially expressed genes that have FDR P values of 0.05 or less. Ingenuity Pathway Analysis (IPA) was performed with default parameters for determining enrichment of canonical pathways. Gene Ontology (GO) analysis was performed with CLC Genomics Workbench 12 with go_association_goslim_20131027 dataset.

### Statistical analysis

Student t-tests were used to evaluate the significance of changes in all combination treatment assays compared to controls. Data collected from each experiment was used to calculate the mean values and standard deviations (SD). Experiments were repeated three times independently. Differences were considered statistically significant if P < 0.05.

## SUPPLEMENTARY MATERIALS










