# Supplementary material for: A novel curcumin analog inhibits canonical and non-canonical functions of telomerase through STAT3 and NF-κB inactivation in colorectal cancer cells
Source: Oncotarget. 2019 Jul 16;10(44):4516–31. doi: 10.18632/oncotarget.27000 (PMC6642039; doi:10.18632/oncotarget.27000)
Supplement: Supplementary file 1 [file oncotarget-10-4516-s001.pdf]

A novel curcumin analog inhibits canonical and non-canonical functions of telomerase through STAT3 and NF-κB inactivation in colorectal cancer cells

SUPPLEMENTARY MATERIALS

Supplementary Table 1: Ingenuity Pathway Analysis (IPA) data of HCT116 treated with compound 19
See Supplementary Table 1

Supplementary Table 2: Gene Ontology (GO) analysis data of HCT116 treated with compound 19
See Supplementary Table 2

Supplementary Table 3: Ingenuity Pathway Analysis (IPA) data of DLD1 treated with compound 19
See Supplementary Table 3

Supplementary Table 4: Gene Ontology (GO) analysis data of DLD1 treated with compound 19
See Supplementary Table 4
